# Mobile app-based cognitive decision-making and memory games enhance cognitive function in older adults with mild cognitive impairment

**DOI:** 10.3389/fpsyg.2025.1633043

**Published:** 2025-10-09

**Authors:** Ampha Pumpho, Rumpa Boonsinsukh, Kitiyawadee Srisim, Supapon Kaewsanmung, Phannarin Suwannarat, Petcharat Keawduangdee

**Affiliations:** ^1^School of Integrative Medicine, Mae Fah Luang University, Chiang Rai, Thailand; ^2^Faculty of Physical Therapy, Srinakharinwirot University, Nakhon Nayok, Thailand

**Keywords:** working memory, mobile, game, application, cognitive training

## Abstract

**Background:**

In present-day society, interactive mobile devices are being more frequently used to bolster the memory capacities of senior individuals with mild cognitive impairment (MCI). However, there is a lack of cognitive training mobile game applications that utilize image processing technologies to assess player behaviors concerning cognitive abilities such as executive functioning, memory retention, discrimination and decision-making, and processing speed.

**Objective:**

The purpose of this study was to develop a mobile gaming app and investigate whether cognitive-motor training mobile game applications that use image processing to recognize player actions in relation to cognitive abilities like executive functioning, memory, discrimination, and decision making would improve cognitive performances in older adults with MCI.

**Methods:**

We performed a randomized controlled trial of a mobile game app cognitive training group and control group in 42 older adults with mild MCI. The intervention group (*n* = 21) spent 30 min/day, 2 days/week for 4 weeks, using the mobile app’s cognitive training game. The control group (*n* = 21) did not receive any training. All the participants were assessed with the neuropsychological tests before and after training.

**Results:**

Following the training, the Training group exhibited significantly greater improvements in Stroop test performance, including increased correct responses (*p* = 0.02, Cohen’s *d* = 0.71) and reduced uncorrected errors (*p* = 0.04, Cohen’s *d* = −0.67). Moreover, auditory reaction time was significantly enhanced (*p* = 0.03, Cohen’s *d* = −0.34) compared with the control group, indicating moderate effect sizes and suggesting clinically meaningful benefits of the intervention.

**Conclusion:**

A newly developed mobile gaming application is a potential tool for training executive function in older adults with MCI.

## Introduction

Globally, life expectancy is increasing. By 2030, 1 in 6 people in the world will be 60 years of age or older (World Health Organization, 2022). As our societies age, the worldwide estimate of people with dementia has reached over 50 million, and that number will almost triple by 2050 (World Health Organization, 2021). Mild cognitive impairment (MCI) is a transitional state between normal cognition and dementia, and it is the best predictor of future dementia including Alzheimer’s disease (Tangalos and Petersen, 2018). For individuals with already existing cognitive impairment, maintaining or improving cognitive function is an important goal. Consequently, several studies investigated how effective various cognitive interventions benefit older adults with mild cognitive impairment on their preserved cognitive abilities (Chaikham et al., 2016; Finn and McDonald, 2011; Rodakowski et al., 2015). The main two approaches of training older persons in cognitive ability that have been successfully implemented are strategy training and extended practice. A “top down” approach, strategy training has been used for enhanced reasoning, memory, complex planning skill. Extended practice, which employs a “bottom up” strategy, has been used for training attention, discriminating, memory, and dual-task performance (Zelinski, 2009).

Game-based interventions for cognitive training have demonstrated encouraging results in the elderly with mild cognitive impairment (MCI). Several research studies have evaluated digital game prototypes such as EmoGame, tele-Exergame, and COGNIPLAT (Damayanti and Ali, 2023; Goumopoulos et al., 2023; Park et al., 2022). These interventions are designed to enhance cognitive functions, memory, and attention, while also addressing emotional and cognitive challenges commonly found in older individuals with MCI. EmoGame primarily focuses on emotional recognition and social cognition training through gamified interactive tasks, while COGNIPLAT provides a comprehensive platform for cognitive rehabilitation, particularly in older adults and patients with neurological disorders. Although these systems have shown promising results, they are often limited by either narrow domain specificity or by the reliance on conventional computerized task designs.

In contrast, the proposed Brain Training mobile game application was designed to integrate multiple cognitive domains with interactive platform through five distinct games (Rabbit Counting, Triangle Counting, Finger Math, Rock–Paper–Scissors, and Direction Memorizing). This design leverages animation, image processing, and interactive problem-solving tasks to simultaneously target attention, working memory, executive function, spatial processing, and processing speed. By combining domain-specific tasks within a highly engaging, mobile-based format, the proposed system aims to provide a more accessible, motivating, and ecologically valid tool for cognitive rehabilitation.

Goal-directed behavior and action (or movement) are crucial to concept of executive function and cognition (Koziol et al., 2012). Interactive cognitive-motor training involves using gross motor movements to engage with computer interface, which participants receive immediate visual feedback via a projection screen and incorporate virtual reality features or exergames. Exergames, or active video gaming, are digital games that require movement of players in order to be engaged in activities, stimulating an active gaming experience to function (Benzing and Schmidt, 2018). Physical exercise appears to cause physiological and metabolic changes that then facilitate specific cognitive activities, particularly executive function through functional adaptations of the brain (Stojan and Voelcker-Rehage, 2019). The parallel information processing, selective attention to task-relevant stimuli, inhibition of task-irrelevant stimuli, and planning/decision-making regarding the motor execution of the response are all required to engage in interactive cognitive-motor training (Schoene et al., 2014).

The process of decision-making is complex and involves assessing a situation, selecting an appropriate plan of action, and evaluating the outcomes (Paulus et al., 2005). In which the “rock, paper, scissors” (RPS) game against computer-simulated pictures of hands was used to investigate how the brain activities during decision-making, prefrontal activity was prominently apparent (Matsubara et al., 2004). Prefrontal activity was more prominent in the “to lose” RPS task than in the “to win” RPS task, as “to lose” RPS requires inhibition of behavior that might increase prefrontal activity (Kikuchi et al., 2007).

Nowadays, the use of smartphone had steadily increased. Prediction between 2024 and 2029 stated the number of smartphone users worldwide will rise continuously by 1.5 billion users, or 30.6 percent. In 2029 the number of smartphone users is expected to reach 6.4 billion (Statista, 2024). A rise in the number of research has investigated the feasibility of cognitive function training on digital platforms. By combining elements of fun and user engagement into their design, “serious games” with goals beyond pure entertainment, such as training, communication, assessment, or improving cognitive and physical health, have recently gained more attention from researchers due to their potential to improve sustained participation in ongoing assessment and therapy (Kim et al., 2015; Lumsden et al., 2016). The cognitive games aim to stimulate mental activity related to memory, calculation, and perception. This could may include leisure computer games as well as those designed specifically for enhancing mental capacity (Niederstrasser et al., 2016). Recent studies have consistently shown that playing games can improve several of cognitive abilities, including working memory, visual-spatial perception, and executive functions (Nobakht et al., 2020).

Presently, modern mobile devices with built-in sensors such as accelerometer, gyroscope, and magnetometer can detect how users interact with games, their movement and surrounding (Intarasirisawat et al., 2019). Nowadays, interactive mobile devices are used for therapy to rehabilitation of the elderly with MCI. However, there is a lack of cognitive training mobile game applications that use image processing to recognize player actions in relation to cognitive abilities like executive functioning, memory, discrimination, and decision making. To facilitate the cognitive performances, we aimed to design an interactive cognitive-motor game-based training that is applicable on mobile devices. We developed mobile game app that included: “to win” or “to lose” Rock-Paper-Scissor, Finger Math, Rabbit Counting, Triangle Counting, and Direction memorizing game. Our objectives were; (a) an easy user interface for elderly people, (b) to design an interactive cognitive-motor training, (c) data analysis for determine whether a 4-week interactive cognitive-motor training using a mobile application may enhance cognitive functions (especially working memory, attention, executive function, and processing speed) of older persons with MCI.

## Materials and methods

### Study design

This study was designed as a randomized controlled trial to investigate the efficacy of a mobile game based cognitive training intervention. The core hypothesis was that repeated engagement with the specially designed game applications (Rabbit Counting, Triangle Counting, Finger Math, Rock–Paper–Scissors, and Direction Memorizing) would enhance cognitive performance by targeting key domains such as working memory, attention, processing speed, and executive function. Specifically, it was hypothesized that participants assigned to the training group would demonstrate greater improvements in cognitive outcomes compared to those in the control group.

The independent variable was the mobile game–based cognitive training (experimental condition) and the control condition. The dependent variables were the cognitive performance which assessed by using the standardized neuropsychological tests: the Digit Span Forward and Backward Test (working memory), the Stroop test (attention and executive control), and reaction time tasks (processing speed).

The rationale for this design is based on the assumption that the interactive and adaptive nature of the mobile games requires continuous engagement of core cognitive processes. For example, the Rabbit Counting and Triangle Counting games are expected to stimulate sustained attention and visual working memory, while the Finger Math game integrates arithmetic problem-solving with visuomotor coordination, thereby engaging executive function and working memory simultaneously. The Rock–Paper–Scissors game was designed to require rapid decision-making, inhibitory control, and motor responses, thus stimulating executive function and processing speed. In addition, the Direction Memorizing game requires participants to encode, retain, and reproduce sequences of directional cues, thereby targeting spatial working memory and attentional control. Through repeated practice in a game-based environment, these tasks are expected to induce measurable improvements in the corresponding cognitive domains.

### Randomization and concealed allocation

The utilization of a randomization table generated by a computer software application was employed to implement a simple randomization with an allocation ratio of 1:1. The sequence of allocation was concealed from the researcher who enrolled and evaluated participants through sequentially numbered, opaque, sealed, and stapled envelopes.

### Participants

The participants were recruited through advertisements in the community service station. The following inclusion criteria were used to recruit the participants: (1) community-dwelling individuals aged 60–80 years, (2) a score of less than 25 on the Thai version of the Montreal Cognitive Assessment (MoCA), and (3) utilize a mobile phone with Android operating system. Subjects were excluded if they had (1) neurological disorders such as stroke, Parkinson’s disease, (2) hearing loss, and (3) severe visual impairment.

**FIGURE 1:**
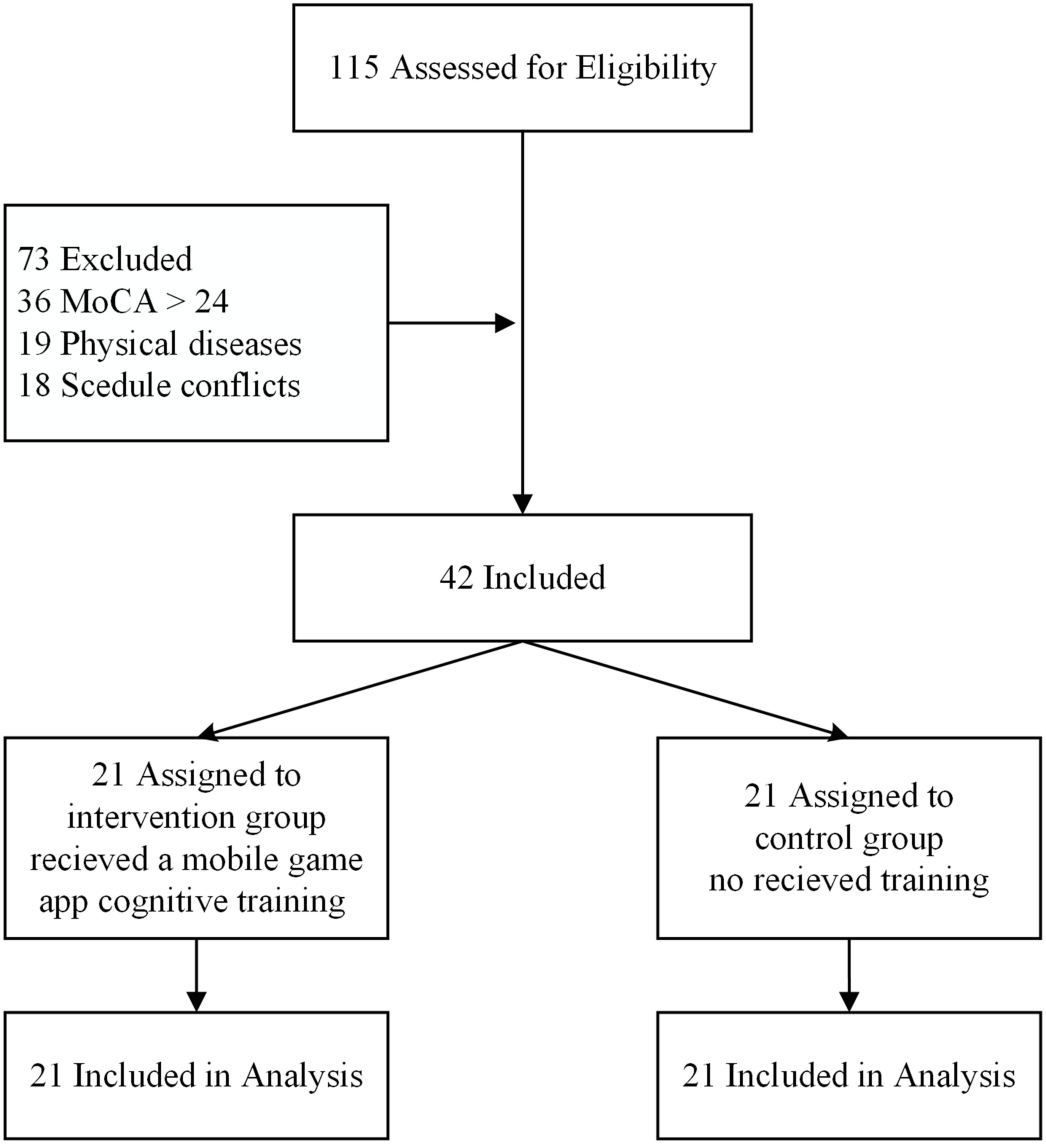
Flow chart showing the number of participants in each group.

A voluntary group of 115 older adults from the community was contacted. We excluded 73 older adults who (1) MoCA-Thai score of more than 24 (*n* = 36), (2) had neurological disorders or vision or hearing impairments (*n* = 19), or (3) could not guarantee a full participation due to scheduling conflicts (*n* = 18). Thus, 42 participants were randomly assigned to either a mobile game app cognitive training group (intervention group, *n* = 21) or a control group (no training, *n* = 21). Figure 1 shows the flowchart of participants throughout the study.

### Procedure

Cognitive assessments were given to all of the participants in these two groups both before and after the training. Assessors administering cognitive tests were blind to the training or no training assignment. After baseline assessment, individuals in the intervention group received 8 training sessions over the duration of about 4 weeks. There were two training sessions each week, lasting about 30 min per session.

### Interventions

**FIGURE 2:**
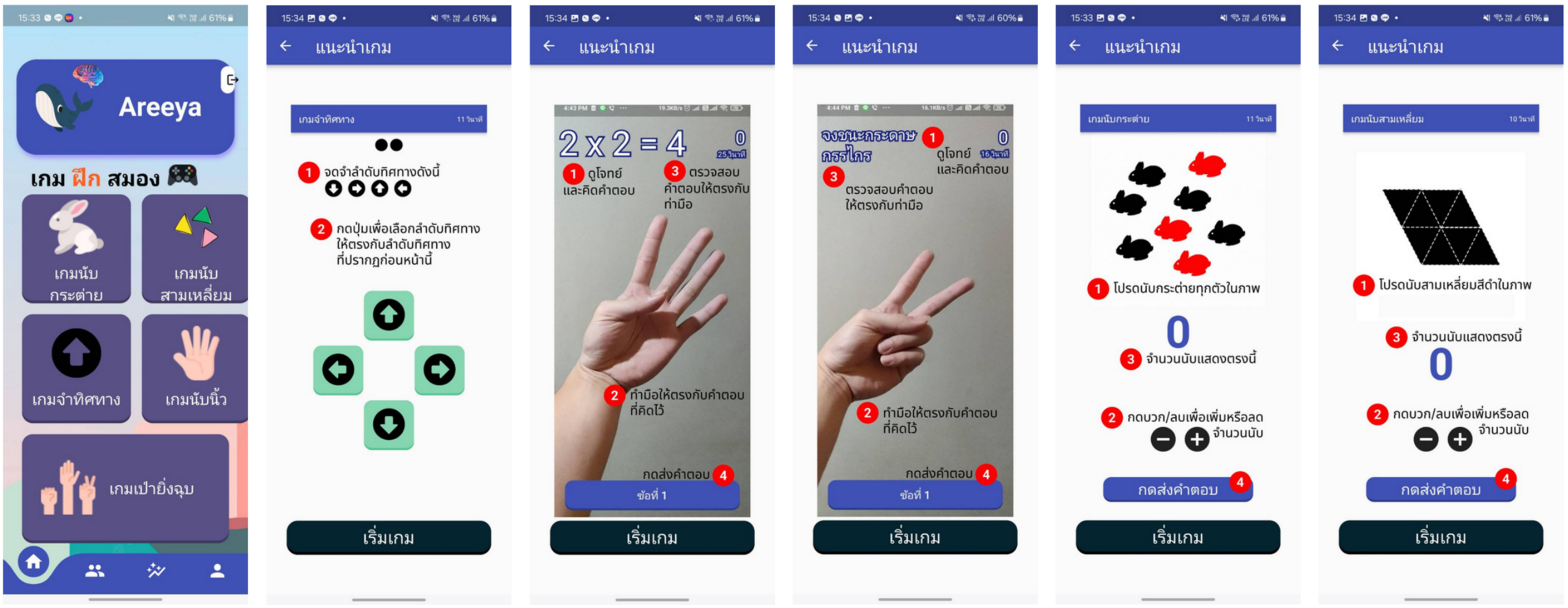
Mobile game application “Brain training”.

We developed a mobile application (Brain Training) that operates on Android devices version 7 or higher (Figure 2). The mobile game application was designed for easy interaction with users and facilitate executive and cognitive functions. This app includes five games: “to win” or “to lose” Rock-Paper-Scissor, Finger Math, Rabbit Counting, Triangle Counting, and Direction Memorizing game. Table 1 provides an overview detailed information of each game.

**TABLE 1:** Overview detailed information of each game in the “Brain Training” application delivered to the intervention group.

Game	Cognitive domain	Task
Finger math	Executive function and working memory	The participants practiced on addition, multiplication, and subtraction problems using Arabic numbers in this game. The player moves their hand to indicate the number of the answer in order to respond to the question scenario. Accuracy was assured by giving the player direct feedback and the chance to resolve the problem if their initial answer was incorrect.
Rabbit counting	Attention and visual working memory	The task assigned to the participants was counting the total number of rabbits that appeared in red and black. They were prompted to press the + or – buttons to adjust the number and submit their response.
Triangle ccounting	Attention and visual working memory	Participants count adjacent triangle boxes and press the + or – buttons to adjust the number and submit their response.
Direction memorizing	Attention and spatial working memory	The participants were asked to recall the on-screen direction signs. Next, they press the direction sign button according to the sequence in which it appears.
Rock-paper-scissors	Executive function and processing speed	The rules for playing the game “rock-paper-scissors”, where “paper” beats “rock”, “rock” beats “scissors”, and “scissors” beats “paper”. The game will display the scenario in which the participants must win or lose against “rock”, “paper”, or “scissor”, the participants move their hand to demonstrate their answer.

#### Direction memorizing game

The development process of the Direction Memorizing game system involved designing visual stimuli using .jpg image files. These images were created with Canva, a graphic design software. To enhance the system’s functionality, a Flutter plugin called “sensors” was integrated; this plugin provides access to the device’s accelerometer, gyroscope, and magnetometer sensors, which are utilized within the application for motion detection and interaction.

The game’s response verification mechanism relies on comparing the user’s indicated direction with the target direction presented in each question. When the user’s response matches the specified direction, a vibration alert is triggered to provide haptic feedback. Upon completion of all questions, the total score is displayed on the summary screen.

The gameplay involves presenting images depicting different directional cues. Each round consists of five images, with each image randomly selected from a database to ensure variability. Before each response, the direction to be matched is displayed, and the participant responds by indicating the corresponding direction. Correct responses are confirmed by a vibration signal, informing the user that their response has been registered successfully.

#### Rock-paper-scissor and finger math game

The development of the Rock-Paper-Scissor and Finger Math game involved the implementation of a custom image processing model to facilitate real-time hand gesture recognition. The process commenced with construction of an image dataset comprising 70 images each of rock, scissors, and paper, totaling 210 images, which was subsequently uploaded to the Roboflow platform for image annotation targeting the region of interest. The annotated dataset was then exported in the form of a TensorFlow TFRecord file, serving as the input for training an SSD MobileNet object detection model. Upon successful training, the resulting model was converted into a TensorFlow Lite (.tflite) format to support deployment within the mobile application.

To enable seamless integration of the image recognition capabilities, the application required the installation of the TensorFlow Lite plugin, a Flutter-based plugin that provides access to the TensorFlow Lite API. This facilitates object detection and image classification with SSD MobileNet model, thereby supporting real-time recognition tasks on mobile devices.

The Rock-Paper-Scissor gameplay involves presenting users with a prompt, which indicates whether the player should win or lose the round and then waits for a user’s hand gesture as rock, paper, or scissors to be recognized as the answer of the round. Each round comprises ten randomly selected questions retrieved from a well-prepared database. During gameplay, the system leverages the device’s camera to capture users’ hand gestures and employs the trained model to predict the corresponding hand sign. The predicted output is then compared with the prompt’s requirement. Correct responses are signified by the appearance of a check mark for one second, while incorrect responses trigger a cross mark and a brief vibration, providing immediate visual and haptic feedback.

The Finger Math game was designed to facilitate cognitive training through interactive numerical tasks. In each session, participants were presented with 10 arithmetic problems, each randomly retrieved from a database to ensure variability across trials. Responses were captured using image processing technology. Specifically, the mobile device’s camera was activated to allow participants to respond by displaying hand gestures. The SSD MobileNet Model trained from an image dataset comprising 70 images each of zero, one, two, three, four and five hand gestures, totaling 420 images, was used for analyzing and predicting the numerical value represented. When the numerical predicted value corresponded to the intended response, participants confirmed their answer by pressing a button located at the bottom of the screen.

#### Rabbit counting game

The Rabbit Counting game was developed to provide a cognitive training tool through interactive gameplay. The animation of game elements was created using Photoshop by accessing the Window menu and selecting Timeline to generate the required motion sequences. A basic comparison function was implemented to evaluate participants’ responses; when the selected answer matched the correct result, the score was automatically increased. At the end of each session, the cumulative score was presented on a summary page.

During gameplay, participants were instructed to count the number of rabbits displayed in each image. A total of ten images were presented per session, with each image randomly retrieved from a database. All images were formatted as .gif files to provide animated visualization, thereby enhancing engagement and maintaining attention throughout the task.

#### Triangle counting game

The Triangle Counting game was developed as part of a cognitive training program. In this game, participant was presented with a sequence of ten images, each containing a different number of triangles. The images were randomly retrieved from a database to ensure variability during each session. Each image was displayed for 5 s, after which the screen automatically switched to a countdown timer before proceeding to the next image.

All images were designed using Canva and stored in .jpg format. Randomization of tasks was implemented using a standard looping algorithm without the use of additional plug-ins. A simple comparison function was employed to evaluate the participants’ responses. When a response matched the correct result, the score was increased accordingly. At the end of each session, the cumulative score was displayed on a summary page.

The application generated a summary of each player’s score and also the speed of responses. Upon completion of a session, participants had the option to initiate a new game using the replay function. Furthermore, the system incorporated a performance-tracking feature that displayed graphical representations of individual progress across daily, weekly, and monthly timeframes. This design allowed for continuous monitoring of cognitive training outcomes and facilitated long-term engagement with the intervention.

### Outcome measures

Interviews with each participant were used to gather baseline information, such as age, gender, underlying condition, and educational attainment. The forward and backward digit span test, Stroop test, and reaction time test were used to evaluate the cognitive performances.

#### Digit span forward and digit span backward

The forward and backward span tests are one of the most frequently neuropsychological assessments for measure short-term verbal memory among older adults (Woods et al., 2011). The forward digit span test required participants to repeat the numbers in a forward order, while the digit span backward test required them to recall the numbers in reverse order. Beginning with a two-number sequence, each correctly repeated series was followed by a sequence with one extra digit. For each sequence, if the participants failed the first time, they were given a second chance using a different set of random numbers. If they failed on the second attempt, the test was stopped, and the longest series was scored (Leung et al., 2011).

#### Stroop color-word test

The term of “executive function” refers to the capacity of shifting of mental sets, monitoring and updating of working memory representation, and inhibition of prepotent responses (Miyake et al., 2000). The Stroop color naming task is expected to stimulate executive functions because participants must act against their usual tendencies (e.g., name the ink color of the word instead of reading the word) (Kofman et al., 2006). The correct answers achieved in the first 60 s was counted. The uncorrected errors were also recorded.

#### Reaction time test

Many cognitive processes require an efficient, sufficient information processing speed to complete relevant tasks in a constrained amount of time (Williams et al., 1996). Thus, response speed test that measure processing speed directly are useful in determining the nature of related attention deficit (van Breukelen et al., 1995). For reaction time assessment in this study, the auditory and visual reaction time were recorded by the Multi Choice Reaction Timer (Mahidol University). The participants were instructed to take a comfortable chair and place their forearms on the desk. In addition, the participants were instructed to place their finger lightly on the reaction timer’s microswitch key and to prepare to press it as quickly as possible in response to either visual stimulus of seeing the red light on the screen or the auditory stimulus of hearing it.

### Statistical analysis

The *t*-test has been used to compare the baseline characteristics of the control group and intervention group for continuous variables. The Chi-square test was used to compare the gender between these groups. Paired *t*-tests were utilizing to determine whether the outcome variables changed within the intervention or control group. The outcomes between the intervention group and control group were compared using Independent *t*-tests. IBM SPSS version 20.0 software was utilized for conducting statistical analyses. Significance was determined at *p* < 0.05.

## Results

Figure 1 shows the flow of participants. The age, gender, years of education, and MoCA-Thai score of the participants in these two groups were not differ significantly (as seen in Table 2). The baseline scores for each cognitive performance outcome revealed no significant different between the training group and the control group.

**TABLE 2:** Baseline characteristics of both groups.

Demographic variables	Intervention (n = 21)	Control (n = 21)	*p*-value
Age, *y*	67.05 ± 4.36	66.52 ± 1.02	0.71
Sex			0.12
Female (%)	19 (90.5%)	15 (71.4%)	
Male (%)	2 (9.5%)	6 (28.6%)	
Education, *y*	5.90 ± 0.44	6.29 ± 0.90	0.89
MoCA score	19.43 ± 4.81	19.14 ± 4.08	0.84

The results of within group comparison showed that the cognitive mobile app training group had significantly increased auditory reaction time (*p* < 0.01), increased in the number of correctly responses (*p* < 0.01), and decreased the uncorrected errors (*p* < 0.01) in Stroop test (as seen in Table 3). Whereas there was no significant difference in the control group (as seen in Table 4).

**TABLE 3:** Comparison within intervention group.

Neuropsychological assessment	Pre-test	Post-test	*p*-value	Cohen D effect size
Digit span forward	5.48 ± 1.25	5.62 ± 1.07	0.48	−0.16
Digit span backward	2.19 ± 0.93	2.14 ± 0.91	0.33	0.22
**Stroop test**				
Correct answer	13.62 ± 4.19	15.83 ± 3.40	<0.01**	−1.11
Uncorrected errors	5.33 ± 4.14	3.31 ± 3.39	<0.01**	1.04
**Reaction time**				
Auditory reaction time	0.65 ± 0.26	0.45 ± 0.13	<0.01**	0.65
Visual reaction time	0.46 ± 0.15	0.44 ± 0.11	0.55	0.13

**Depict a significant difference at *p* < 0.01.

**TABLE 4:** Comparison within control group.

Neuropsychological assessment	Pre-test	Post-test	*p*-value	Cohen D effect size
Digit span forward	5.57 ± 1.29	5.90 ± 0.89	0.09	−0.39
Digit span backward	2.48 ± 0.87	2.38 ± 0.74	0.54	0.14
**Stroop test**				
Correct answer	13.00 ± 5.55	12.74 ± 5.10	0.54	0.14
Uncorrected errors	6.00 ± 5.59	6.21 ± 5.09	0.64	−0.10
**Reaction time**				
Auditory reaction time	0.62 ± 0.34	0.52 ± 0.27	0.12	0.36
Visual reaction time	0.50 ± 0.15	0.45 ± 0.11	0.08	0.41

**FIGURE 3:**
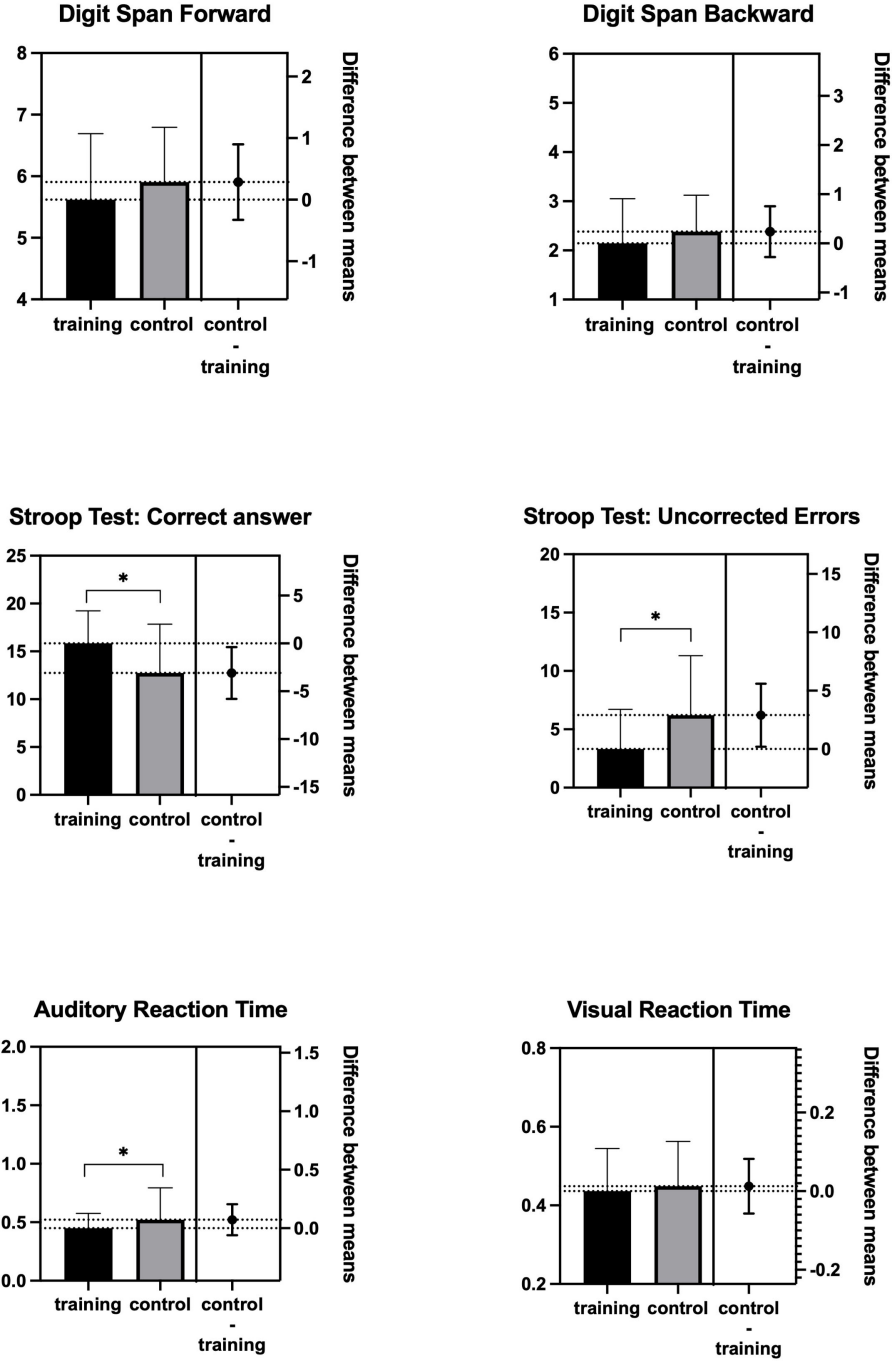
Comparison between training group and control group.

Following the training, the Training group exhibited significantly greater improvements in Stroop test performance, including increased correct responses (*p* = 0.02, Cohen’s *d* = 0.71) and reduced uncorrected errors (*p* = 0.04, Cohen’s *d* = −0.67). Moreover, auditory reaction time was significantly enhanced (*p* = 0.03, Cohen’s *d* = −0.34) compared with the control group (as seen in Table 5 and Figure 3).

**TABLE 5:** Comparison between groups after training.

Neuropsychological assessment	Intervention (n = 21)	Control (n = 21)	*p*-value	Cohen D effect size
Digit span forward	5.62 ± 1.07	5.90 ± 0.89	0.35	−0.29
Digit span backward	2.14 ± 0.91	2.38 ± 0.74	0.36	−0.29
**Stroop test**				
Correct answer	15.83 ± 3.40	12.74 ± 5.10	0.02*	0.71
Uncorrected errors	3.31 ± 3.39	6.21 ± 5.09	0.04*	−0.67
**Reaction time**				
Auditory reaction time	0.45 ± 0.13	0.52 ± 0.2	0.03*	−0.34
Visual reaction time	0.44 ± 0.11	7 0.45 ± 0.11	0.73	−0.11

*Depict a significant difference at *p* < 0.05.

## Discussion

To our knowledge this is the first study to examine the effect of cognitive training mobile game applications that use image processing to recognize player response actions in low educated elderly with MCI.

We developed and designed the mobile application named “Brain Training,” facilitating easy interaction with users to record both accurate and inaccurate responses during the decision-making and memory game sessions. We also investigated the effect of mobile application-based cognitive training on cognitive functions in older adults with Mild Cognitive Impairment (MCI). The results of our study support our hypotheses, and provide empirical evidence regarding the impact of cognitive training via mobile applications on the cognitive functions.

The results revealed significant differences in changes in performance on the Stroop test within the intervention group, while the control group showed no significant differences. The training group exhibited significantly greater improvements in Stroop test performance, including increased correct responses and reduced uncorrected errors.

Our findings were in agreements with previous study that reported the effects of cognitive training on Stroop performances. The study by Hwang et al. (2012) revealed the effectiveness of cognitive training in individuals with Mild Cognitive Impairment (MCI). The findings exhibited a significant tendency toward improved accuracy in color recognition during the Stroop test at the 3-month follow-up as compared to the baseline evaluation (Hwang et al., 2012). Additionally, a study conducted by Husseini et al. (2016) demonstrated the effectiveness of a computerized cognitive training program in enhancing performance on the Stroop task by reducing error number in elderly women with MCI (Husseini et al., 2016). The possible explanation may arise from the incorporation of the Rock-Paper-Scissors (RPS) game in our mobile gaming application. Research indicates that engagement in the RPS game has been shown to activate various areas of the brain, particularly the prefrontal cortex (PFC), a region known for its role in executive cognitive functions such as decision-making, inhibitory control, and cognitive flexibility (Kikuchi et al., 2007; Zhou, 2016).

We hypothesized that engaging in cognitive tasks through the utilization of the Finger Math, Rabbit Counting, Triangle Counting, and direction memorizing game could potentially enhance working memory capabilities, as measured by the digit span tasks. The cognitive processes engaged in memory retrieval (or recollection) are complex, involving diverse brain regions and mechanisms (Johnson and Rugg, 2007). Studies have shown that episodic memory recall entails reactivating encoding processes, with neural correlates specific to the type of information remembered. Neuroimaging research has demonstrated that both picture recall and auditory word recall activate a common pathway, including right anterior prefrontal, posterior medial parietal, and possibly bilateral frontal-opercular cortex, requiring to access information learned during a unique event during their episodic memory retrieval tasks (Buckner et al., 1996). However, the results we obtained showed no improvement in task performance for both forward and backward digit span tasks, in contrast to the findings of previous research studies. It is also possible that the training intensity in the present study (two sessions per week over 4 weeks) was insufficient to induce measurable changes in working memory performance. A study by Golino et al. (2017) indicated that adaptive cognitive training, which included 12 individual sessions lasting between 60–90 min once a week, produced a positive impact on performance in the digit span task (Golino et al., 2017). Another study by Ichihara-Takeda et al. (2016) revealed that the ability of participants to correctly recall information from the main session comprising 12 trials of computerized cognitive training exhibited a significantly associated with their performance on the Digit Span backward test, emphasizing the impact of the computerized cognitive task on the functions of working memory.

The present study found significant improvement in auditory reaction time in the training group. The results of this study align with the study by Husseini et al. (2016), which indicated that the computer-based brain training program had a positive impact on enhancing reaction time and processing speed among elderly females with MCI.

### Study limitation

Some limitations are identified within this study. Primarily, the sample size is small, thereby generalization of results is limited. Secondly, the mobile gaming app is exclusively compatible with the Android platform and lacks support for the iOS operating system.

## Conclusion

Our results suggest that the use of cognitive training via mobile game applications in this study has shown to be effective in producing a significant improvement in reaction time and executive functioning especially decision and discrimination making in the elderly with mild cognitive impairment.

## Data Availability

Data supporting the findings of this study are available from the corresponding author upon reasonable request.

## References

[B1] Benzing V. Schmidt M. (2018). Exergaming for children and adolescents: Strengths, weaknesses, opportunities and threats. *J. Clin. Med.* 7:422. 10.3390/jcm7110422 30413016 PMC6262613

[B2] Buckner R. Raichle M. Miezin F. Petersen S. (1996). Functional anatomic studies of memory retrieval for auditory words and visual pictures. *J. Neurosci.* 16 6219–6235. 10.1523/JNEUROSCI.16-19-06219.1996 8815903 PMC6579164

[B3] Chaikham A. Putthinoi S. Lersilp S. Bunpun A. Chakpitak N. (2016). Cognitive training program for thai older people with mild cognitive impairment. *Procedia Environ. Sci.* 36 42–45. 10.1016/j.proenv.2016.09.007

[B4] Damayanti N. Ali N. (2023). Evaluating game application interfaces for older adults with mild cognitive impairment. *Int. J. Adv. Comput. Sci. Appl.* 14 952–956. 10.14569/IJACSA.2023.01406101

[B5] Finn M. McDonald S. (2011). Computerised cognitive training for older persons with mild cognitive impairment: A pilot study using a randomised controlled trial design. *Brain Impair.* 12 187–199. 10.1375/brim.12.3.187

[B6] Golino M. Mendoza C. Golino H. (2017). Effects of cognitive training on cognitive performance of healthy older adults. *Span J. Psychol.* 20:E39. 10.1017/sjp.2017.38 28929999

[B7] Goumopoulos C. Skikos G. Frounta M. (2023). Feasibility and effects of cognitive training with the COGNIPLAT game platform in elderly with mild cognitive impairment: Pilot randomized controlled trial. *Games Health J.* 12 414–425. 10.1089/g4h.2023.0029 37276027

[B8] Husseini F. Damirchi A. Babaei P. (2016). Effect of brain training on cognitive performance in elderly women diagnosed with mild cognitive impairment. *Caspian J. Neurol. Sci.* 2 25–31. 10.18869/acadpub.cjns.2.7.25

[B9] Hwang H. Choi S. Yoon D. Yoon B. Yoon B. Suh Y. (2012). The effect of cognitive training in patients with mild cognitive impairment and early Alzheimer’s disease: A preliminary study. *J. Clin. Neurol.* 8 190–197. 10.3988/jcn.2012.8.3.190 23091528 PMC3469799

[B10] Ichihara-Takeda S. Takeda K. Ikeda N. Matsuyama K. Funahashi S. (2016). Neuropsychological assessment of a new computerized cognitive task that was developed to train several cognitive functions simultaneously. *Front. Psychol.* 7:497. 10.3389/fpsyg.2016.00497 27148110 PMC4828453

[B11] Intarasirisawat J. Ang C. Efstratiou C. Dickens L. Page R. (2019). “Exploring the Touch and Motion Features in Game-Based Cognitive Assessments,” in *Proceedings of the ACM on interactive, mobile, wearable and ubiquitous technologies*, Vol. 3 (New York, NY: ACM), 1–25.

[B12] Johnson J. Rugg M. (2007). Recollection and the reinstatement of encoding-related cortical activity. *Cereb. Cortex* 17 2507–2515. 10.1093/cercor/bhl156 17204822

[B13] Kikuchi S. Iwata K. Onishi Y. Kubota F. Nisijima K. Tamai H. (2007). Prefrontal cerebral activity during a simple “rock, paper, scissors” task measured by the noninvasive near-infrared spectroscopy method. *Psychiatry Res. Neuroimaging* 156 199–208. 10.1016/j.pscychresns.2007.01.002 17976959

[B14] Kim K. Choi Y. You H. Na D. Yoh M. Park J. (2015). Effects of a serious game training on cognitive functions in older adults. *J. Am. Geriatr. Soc.* 63 603–605. 10.1111/jgs.13304 25800915

[B15] Kofman O. Meiran N. Greenberg E. Balas M. Cohen H. (2006). Enhanced performance on executive functions associated with examination stress: Evidence from task-switching and Stroop paradigms. *Cogn. Emot.* 20 577–595. 10.1080/02699930500270913

[B16] Koziol L. Budding D. Chidekel D. (2012). From movement to thought: Executive function, embodied cognition, and the cerebellum. *Cerebellum* 11 505–525. 10.1007/s12311-011-0321-y 22068584

[B17] Leung J. Lee G. T. H. Lam Y. H. Chan R. C.-K. Wu J. Y. M. (2011). The use of the Digit Span Test in screening for cognitive impairment in acute medical inpatients. *Int. Psychogeriatr.* 23 1569–1574. 10.1017/S1041610211000792 21729426

[B18] Lumsden J. Edwards E. Lawrence N. Coyle D. Munafò M. (2016). Gamification of cognitive assessment and cognitive training: A systematic review of applications and efficacy. *JMIR Serious Games* 4:e5888. 10.2196/games.5888 27421244 PMC4967181

[B19] Matsubara M. Yamaguchi S. Xu J. Kobayashi S. (2004). Neural correlates for the suppression of habitual behavior: A functional MRI study. *J. Cogn. Neurosci.* 16 944–954. 10.1162/0898929041502643 15298782

[B20] Miyake A. Friedman N. Emerson M. Witzki A. Howerter A. Wager T. (2000). The unity and diversity of executive functions and their contributions to complex “Frontal Lobe” tasks: A latent variable analysis. *Cogn. Psychol.* 41 49–100. 10.1006/cogp.1999.0734 10945922

[B21] Niederstrasser N. Hogervorst E. Giannouli E. Bandelow S. (2016). Approaches to cognitive stimulation in the prevention of dementia. *J. Gerontol. Geriatr. Res.* S5:5. 10.4172/2167-7182.S5-005

[B22] Nobakht F. Mirmahdi R. Heidari H. (2020). Effect of computer games (puzzle game and Simulation game) in working memory and space visual perception in student with specific learning disorder (reading, writing, math). *Arch. Pharma Pract.* 11 55–59.

[B23] Park C. Mishra R. York M. Enriquez A. Lindsay A. Barchard G. (2022). Tele-medicine based and self-administered interactive exercise program (Tele-Exergame) to improve cognition in older adults with mild cognitive impairment or dementia: A feasibility, acceptability, and proof-of-concept study. *Int. J. Environ. Res. Public Health* 19:16361. 10.3390/ijerph192316361 36498431 PMC9739527

[B24] Paulus M. Feinstein J. Leland D. Simmons A. (2005). Superior temporal gyrus and insula provide response and outcome-dependent information during assessment and action selection in a decision-making situation. *Neuroimage* 25 607–615. 10.1016/j.neuroimage.2004.12.055 15784440

[B25] Rodakowski J. Saghafi E. Butters M. Skidmore E. (2015). Non-pharmacological interventions for adults with mild cognitive impairment and early stage dementia: An updated scoping review. *Mol. Aspects Med.* 43 38–53. 10.1016/j.mam.2015.06.003 26070444 PMC4600436

[B26] Schoene D. Valenzuela T. Lord S. de Bruin E. D. (2014). The effect of interactive cognitive-motor training in reducing fall risk in older people: A systematic review. *BMC Geriatr.* 14:107. 10.1186/1471-2318-14-107 25240384 PMC4181419

[B27] Statista. (2024). *Number of smartphone users worldwide from 2014 to 2029.* Available online at: https://www.statista.com/forecasts/1143723/smartphone-users-in-the-world (accessed March 12, 2024)

[B28] Stojan R. Voelcker-Rehage C. (2019). A systematic review on the cognitive benefits and neurophysiological correlates of exergaming in healthy older adults. *J. Clin. Med.* 8:734. 10.3390/jcm8050734 31126052 PMC6571688

[B29] Tangalos E. Petersen R. (2018). Mild cognitive impairment in geriatrics. *Clin. Geriatr. Med.* 34 563–589. 10.1016/j.cger.2018.06.005 30336988

[B30] van Breukelen G. Roskam E. Eling P. Jansen R. Souren D. Ickenroth J. G. M. A. (1995). model and diagnostic measures for response time series on tests of concentration: Historical background, conceptual framework, and some applications. *Brain Cogn.* 27 147–179. 10.1006/brcg.1995.1015 7772331

[B31] Williams M. LaMarche J. Alexander R. Stanford L. Fielstein E. Boll T. (1996). Serial 7s and Alphabet backwards as brief measures of information processing speed. *Arch. Clin. Neuropsychol.* 11 651–659. 10.1016/S0887-6177(96)80002-3

[B32] Woods D. Kishiyama M. Yund E. Herron T. Edwards B. Poliva O. (2011). Improving digit span assessment of short-term verbal memory. *J. Clin. Exp. Neuropsychol.* 33 101–111. 10.1080/13803395.2010.493149 20680884 PMC2978794

[B33] World Health Organization. (2021). *Dementia.* Available online at: https://www.who.int/news-room/facts-in-pictures/detail/dementia (accessed December 25, 2023)

[B34] World Health Organization. (2022). *Aging and health.* Available online at: https://www.who.int/news-room/fact-sheets/detail/ageing-and-health (accessed December 24, 2023)

[B35] Zelinski E. (2009). Far transfer in cognitive training of older adults. *Restor. Neurol. Neuros.* 27 455–471. 10.3233/RNN-2009-0495 19847070 PMC4169295

[B36] Zhou H. (2016). The rock-paper-scissors game. *Contemp. Phys.* 57 151–163. 10.1080/00107514.2015.1026556

